# Establishing Genotype-to-Phenotype Relationships in Bacteria Causing Hospital-Acquired Pneumonia: A Prelude to the Application of Clinical Metagenomics

**DOI:** 10.3390/antibiotics6040030

**Published:** 2017-11-29

**Authors:** Etienne Ruppé, Abdessalam Cherkaoui, Vladimir Lazarevic, Stéphane Emonet, Jacques Schrenzel

**Affiliations:** 1 Genomic Research Laboratory, Geneva University Hospitals, CMU-9F, Rue Michel Servet 1, CH-1211 Geneva 14, Switzerland; vladimir.lazarevic@genomic.ch (V.L.); jacques.schrenzel@hcuge.ch (J.S.); 2 Laboratory of Bacteriology, University Hospitals, Rue Gabrielle Perret-Gentil 4, CH-1211 Geneva 14, Switzerland; abdessalam.cherkaoui@hcuge.ch; 3 Service of Infectious Diseases, Geneva University Hospitals, Rue Gabrielle Perret-Gentil 4, CH-1211 Geneva 14, Switzerland; stephane.emonet@hcuge.ch

**Keywords:** next-generation sequencing, whole-genome sequencing, hospital-acquired pneumonia, antibiotic resistance, prediction

## Abstract

Clinical metagenomics (CMg), referred to as the application of next-generation sequencing (NGS) to clinical samples, is a promising tool for the diagnosis of hospital-acquired pneumonia (HAP). Indeed, CMg allows identifying pathogens and antibiotic resistance genes (ARGs), thereby providing the information required for the optimization of the antibiotic regimen. Hence, provided that CMg would be faster than conventional culture, the probabilistic regimen used in HAP could be tailored faster, which should lead to an expected decrease of mortality and morbidity. While the inference of the antibiotic susceptibility testing from metagenomic or even genomic data is challenging, a limited number of antibiotics are used in the probabilistic regimen of HAP (namely beta-lactams, aminoglycosides, fluoroquinolones, glycopeptides and oxazolidinones). Accordingly, based on the perspective of applying CMg to the early diagnostic of HAP, we aimed at reviewing the performances of whole genomic sequencing (WGS) of the main HAP-causing bacteria (Enterobacteriaceae, *Pseudomonas aeruginosa*, *Acinetobacter baumannii*, *Stenotrophomonas maltophilia* and *Staphylococcus aureus*) for the prediction of susceptibility to the antibiotic families advocated in the probabilistic regimen of HAP.

## 1. Introduction

Clinical metagenomics (CMg) refers to the concept of sequencing the DNA of a clinical sample (without any prior culturing step) with the purpose of recovering clinical information [1]. In the context of the diagnostic of infections, CMg consists in sequencing samples in order to identify putative pathogen(s) and to predict their antibiotic susceptibility profiles. CMg has been applied to an increasing diversity of samples: respiratory samples [2,3,4], urine [5,6], cerebrospinal fluid or brain biopsy [7,8], blood [9,10,11], bone and joint infection samples [12,13,14] and skin granuloma [15]. CMg takes advantages of the recent development of sequencing methods together with bioinformatics tools. So far, most CMg studies have used Illumina-based technology that typically generates millions of reads from 150–300 bp. More recently, long read sequencing methods have been developed (Pacific Biosciences and Oxford Nanopore) but they have barely been used for CMg studies [2,6]. While CMg is a promising approach for the diagnosis of infections, it remains experimental and several hurdles remain to be tackled before it could be used in routine: the removal of the host DNA [2,12,13,16], the capacity to detect pathogens in polymicrobial samples [12,13], the detection of antibiotic resistance genes (ARGs) and other genomic determinants [17], the assessment of the linkage between ARGs and their host in case of polymicrobial samples [12], linking a phenotype to the detected ARGs, the similar turn-around time as compared to conventional, culture-based methods (though it recently tended to decrease, especially when using the Nanopore sequencers [6]), the establishment of consensual quality control markers, the distinction between pathogens and contaminants [18] and not the least, high cost and the reimbursement by healthcare structures. 

While other methods based on real-time PCR are available and may enable the detection of pathogens and of some antibiotic resistance genes (ARGs) [19,20], they include a limited panel of both of them, and do not span mutational events that can be associated with antibiotic resistance. Hence, CMg could overcome these limitations in being able to reconstruct genomes and precisely infer the antibiotic susceptibility profile, possibly before the culture results [6]. Yet, the in silico translation between the genotype into the phenotype may be challenging because it relies on the quality and exhaustiveness of the available knowledge about the genomic determinants of resistance. First, the ARG database needs to be exhaustive so that no ARG shall be missed. Then, the resistance pattern conferred by the ARGs needs to be known, which is sometimes not the case for some variants that have not been experimentally tested. Lastly, many resistance phenotypes arise from mutational events that lead to a decrease affinity of the antibiotic (e.g., mutations in the topoisomerase for fluoroquinolone resistance), an increase of the expression of an intrinsic resistance gene (e.g., *bla*_AmpC_ in Enterobacteriaceae) and/or a decrease of the expression of a gene (e.g., *oprD* in *Pseudomonas aeruginosa*) alone or in combination. Unlike acquired ARGs that have been thoroughly collected, data linking specific mutational events with a resistance phenotype are lacking, thereby introducing some caveats in the genotype-to-phenotype process for some bacteria-antibiotics couples. 

Hospital-acquired pneumonia (HAP) is defined as pneumonia that occur 48 h or more after admission, which was not incubating at the time of admission. Early-onset HAP and VAP are defined as occurring within the first 4 days of hospitalization, while late-onset HAP and VAP occurs beyond 4 days of hospitalization. HAP accounts for up to 25% of all intensive care unit (ICU) infections and for more than 50% of the antibiotics prescribed. he recommended management of HAP relies on the combination of clinical and bacteriological data [21]. When HAP is suspected (upon clinical and radiological grounds), a clinical sample from the lower respiratory tract is collected for quantitative cultures prior to any new antibiotic treatment. The time to antibiotic susceptibility results is usually 48 h, during which a probabilistic antibiotic regimen is given, considering or not the possibility of the presence of resistant bacteria as a causal agent of the pneumonia according to the risk factors of the patient [21]. According to the current guidelines, few antibiotic families are considered for the probabilistic therapy: beta-lactams, aminoglycosides, fluoroquinolones, glycopeptides and oxazolidinones. Indeed at least in immuno-competent patients, HAP is caused by a limited spectrum of bacterial pathogens (possibly more than one), and rarely by viral or fungal pathogens [22]. In early-onset HAP, the most frequently encountered bacteria are Enterobacteriaceae, *Haemophilus* spp. and *Streptococcus pneumoniae*. In other cases, Enterobacteriaceae, *Pseudomonas aeruginosa*, *Acinetobacter baumannii*, and *Staphylococcus aureus* are the most frequent agents. Hence in a genotype-to-phenotype perspective, a limited combination of antibiotics-bacterial species is to be investigated in the context of HAP. To our knowledge, the use of CMg in the context of HAP in immunocompetent patients has been reported once [2], but antibiotic resistance profiles of the pathogens were not investigated [23]. 

In this review, we aimed at reporting the results of the various genotype-to-phenotype studies that have been performed in the main HAP pathogens. We chose to focus on the antibiotic families that are advocated in the probabilistic therapy, before the culture results are available, because we assume that as of now, CMg would be positioned in the HAP context as a rapid test that could allow a rapid adaptation of the probabilistic therapy [2]. Besides, we excluded *S. pneumoniae*, *Haemophilus influenzae*, *Legionella pneumophila* and *Branhamella catarrhalis*, which are occasionally found in HAP but are mostly susceptible to the probabilistic antibiotic options recommended for the HAP treatment. Indeed, provided that they would be correctly identified by CMg, no specific genotype-to-phenotype analysis shall be undertaken with regards to the antibiotics used in the probabilistic treatment of HAP. 

## 2. From Genotype to Phenotype

### 2.1. Protocol

**Figure 1:**
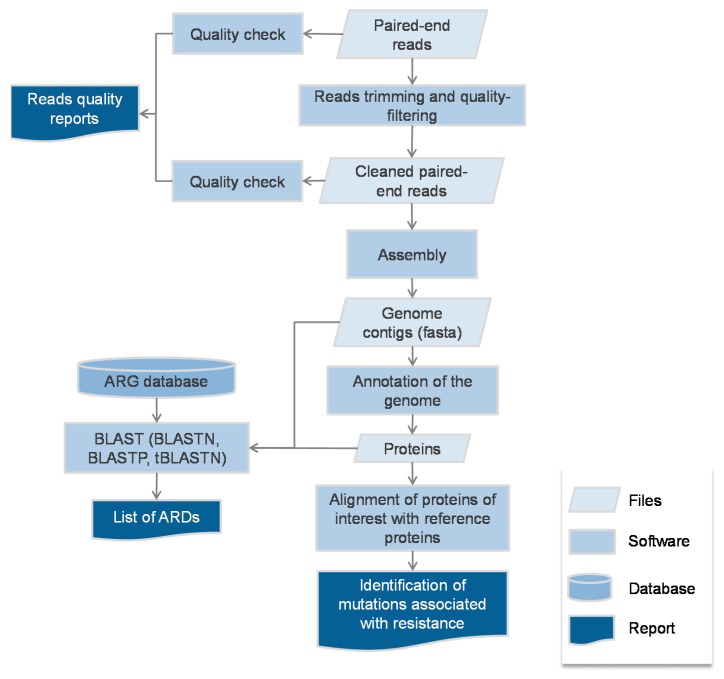
Typical bioinformatics flow-chart of the genotype-to-phenotype studies.

Regarding antibiotic resistance, whole genome sequencing (WGS)-based genotype-to-phenotype studies rely on the detection of ARGs stored in dedicated databases, the most popular being Resfinder [24], CARD [25] and ARG-ANNOT [26] (Figure 1). The ARGs sequences are usually sought using BLAST (BLASTN, BLASTP or tBLASTN [27]), with an identity threshold varying from 80–98% identity over 50–80% of the reference sequence according to the studies. Other studies have used the relative coverage calculated as the product of the identity and the coverage on the reference [28]. Besides alignment based-tools, the Hidden Markov model (HMM)—based tool Resfams [29] has also been used to detect ARGs [30]. Besides the Resfinder, CARD and ARG-ANNOT online search possibilities, some ARG-searching pipelines such as ARIBA (that can be run with any ARG database) [31] or AMR++ (based on the MEGARes database [32]) that enable the detection and counts of ARGs as well as the detection of variants, have been made available. Once identified in the genomic data, an ARG is assumed to be expressed enough to confer resistance to the antibiotics it has been described to provide resistance to. For instance, if a *bla*_CTX-M_ (a gene encoding for a CTX-M type extended-spectrum beta-lactamase (ESBL)) is detected in an *E. coli* genome, the strain shall be considered as resistant to all beta-lactams but co-amoxiclav, piperacillin-tazobactam and carbapenems. Still, the precise antibiotic spectrum of all ARGs found in the databases is not precisely known as only a fraction has been precisely tested, the others being homologues. Hence, some phenotypes are inferred from the phenotype of the closest homologue that has been characterised. For TEM and SHV beta-lactamases, the precise analysis of mutations in the positions known to alter the phenotype (towards to ESBL, resistance to inhibitors or both) has to be performed to infer the spectrum of resistance (see https://www.ncbi.nlm.nih.gov/pathogens/submit-beta-lactamase/). Eventually in NGS-based genotype-to-phenotype studies, the comparator is phenotypic antibiotic susceptibility testing, performed by disk diffusion or broth dilution. Then, three types of results are yielded: correct when WGS agrees with conventional methods, major errors (ME) when WGS predicts resistance while the strain tested susceptible, and very major errors (VME) when WGS predicts susceptibility while the strain tested resistant. 

### 2.2. Escherichia coli

Together with *S. aureus*, most of the genotype-phenotype studies in Enterobacteriaceae have been performed in *E. coli.* [33,34,35]. With regards to the antibiotics used in the probabilistic regimen of HAP, *E. coli* does not harbour any ARG but its chromosomal AmpC-type cephalosporinase [36]. Unlike other AmpC-producing Enterobacteriaceae though, the *E. coli bla*_AmpC_ is not regulated by the AmpD/AmpR system, and has a weak constitutive expression [37]. Nonetheless, specific mutations in the promoter and/or in the upstream regulatory loop can lead to a substantial expression of *bla*_AmpC_and to cephalosporin resistance [38,39], but they are rarely found in clinical isolates. Accordingly, *E. coli* resistance to antibiotics used in the first line of HAP is mainly driven by acquired ARGs, and the accuracy of the prediction rates for antibiotic susceptibility has constantly been high across the three studies: 98.6–100% for ampicillin/amoxicillin, 100% for co-amoxiclav, 97.2–100%, 100% for carbapenems, 97.9–100% for fluoroquinolones and 100% for amikacin [33,34,35] (Table 1). Still, some discrepancies were observed: a *bla*_TEM-1_ harbouring *E. coli* was unexpectedly susceptible to amoxicillin (MIC 6 mg/L) [34]. Likewise, a strain with mutations in the promoting region of *bla*_AmpC_was found to be susceptible to third generation cephalosporins (3GC). Besides, some discrepancies were observed for ceftazidime in strains producing a CTX-M—type ESBL [34], the most frequent ESBL found in clinical isolates. CTX-M ESBLs, which confer a low-level resistance to ceftazidime, and EUCAST advocates considering as susceptible a strain with a ceftazidime MIC ≤ 1 mg/L, whereas in NGS interpretation, a strain harbouring a *bla*_CTX-M_ gene shall be considered as resistant to all 3GC. Another strain had an unexpected resistance to 3GC while no acquired beta-lactamase was detected, and the likely explanation was the presence of an S287R amino acid substitution on AmpC [35]. For fluoroquinolones, the observed VME was explained by the non-consideration of mutational events [33].

### 2.3. Klebsiella pneumoniae

*K. pneumoniae* is intrinsically resistant to penicillins via the production of a narrow-spectrum beta-lactamase (of SHV, LEN of OKP type), which is constitutively expressed [45]. Two studies have focused on the NGS genotype-phenotype correlation in *K. pneumoniae*: Stoesser et al. [34] and a study from our group where some clonal, multidrug-resistant isolates were sequenced [40]. In the study from Stoesser et al. [34], the correct prediction rates for antibiotic susceptibility were high but not as good as for *E. coli*: 98.6% for co-amoxiclav, 97.2% for 3GC, 98.6% for carbapenems, 91.3% for fluoroquinolones and 98.6% for gentamicin (Table 1). As for discrepancies, a strain was unexpectedly found to be susceptible to co-amoxiclav while an oxacillinase-encoding gene (*bla*_OXA-1_) was detected. Also, two strains had mutations in the topoisomerase GyrA but were surprisingly characterized as susceptible to fluoroquinolones. Conversely, respectively two and one strains were found to be resistant to 3GC and meropenem while no acquired beta-lactamase gene that could explain this phenotype was found. In the study from our group [40], 18 multidrug-resistant strains were sequenced. For all the antibiotics considered in the HAP context, a correct prediction was observed in all strains (Table 1). 

### 2.4. Other Enterobacteriaceae Involved in HAP

To our knowledge, no NGS-based genotype-to-phenotype study has been performed for other HAP-causing Enterobacteriaceae (*Citrobacter freundii*, *Citrobacter koseri*, *Enterobacter aerogenes*, *Enterobacter cloacae*, *Hafnia alvei*, *Klebsiella oxytoca*, *Morganella morganii*, *Proteus mirabilis*, *Proteus vulgaris*, *Providencia stuartii*, and *Serratia marcescens*). In a recent study though, Pesesky et al. compared the performance of a rules-based prediction algorithm (such as that used in the other NGS-based genotype-to-phenotype studies) to a logistic regression—based prediction algorithm, using the Hmm-based tool Resfams [30]. They included 78 strains: 34 *E. coli*, 29 *K. pneumoniae*, 9 *E. cloacae* and 6 *E. aerogenes*. While species-level data were not provided, the overall accuracy of the rules-based algorithm was 89.0%, with an ME rate of 6.0% and a VME rate of 4.9%. The logistic regression—based prediction algorithm performed similarly, with an overall accuracy of 90.8%, but with a lower ME rate (2.6%) and a higher VME rate (6.6%). We assume that the prediction of the susceptibility to third-generation cephalosporins (3GC) would be challenging for AmpC-producing Enterobacteriaceae since data about the mutational events leading to its overexpression are lacking. 

### 2.5. Pseudomonas aeruginosas

*P. aeruginosa* intrinsically harbours several resistance determinants: an inducible AmpC-type cephalosporinase [46], an OXA-type carbapenemase (OXA-50, yet not expressed in wild-type strains [47]) and an APH(3′)-IIb (resistance to kanamycin) [48]. In addition, *P. aeruginosa* possesses numerous efflux pumps (MexAB-OprM, MexCD-OprJ, MexEF-OprN, MexXY-OprM, MexJK-OprM and MexGHI-OpmD), of which overexpression can lead to multiple resistances to beta-lactams, aminoglycosides and fluoroquinolones [49], and the porin D2 (encoded by the *oprD* gene), of which loss of expression confers resistance to carbapenems [50]. Indeed, a wide array of mutational events on the *oprD* gene can be observed with possible consequences on the susceptibility to carbapenems [51]. Indeed, the loss of porin D2 in *P. aeruginosa* is associated with meropenem resistance (MIC > 8 µg/mL) in 75% strains, but 25% remaining strains without porin D2 remained in the susceptibility range [51]. Besides, several genetic events (most of them being uncharacterized) can modulate the expression of the latter resistance determinants, making the inference genotype-to-phenotype quite hazardous for antibiotic susceptibility prediction [51]. Consequently, the correct prediction rates (based on the whole genome sequencing of 388 strains [41]) for meropenem (92.4%), levofloxacin (92.8%) and amikacin (81.5%) were lower those observed for *E. coli* and *K. pneumoniae* (Table 1). Clearly, other determinants other than those already associated with resistance in *P. aeruginosa* need to be identified. 

### 2.6. Acinetobacter baumannii

*A. baumannii* is notorious for being involved in hospital-acquired infections including HAP. Like *P. aeruginosa*, *A. baumannii* harbours intrinsic beta-lactamases (a non-inducible AmpC-type cephalosporinase [52] and an OXA-type carbapenemase [OXA-51] [53] that is barely expressed in wild-type strains) and efflux pumps (AdeABC), of which overexpression can lead to antibiotic resistance [54]. Thus as for *P. aeruginosa*, the prediction of antibiotic susceptibility from genomic data shall be challenging since resistance in *A. baumannii*, this can arise from the acquisition of resistance genes (e.g., OXA-23 carbapenemase) and mutational events associated with gene expression such as the insertion of ISAbaI upstream of the *bla*AmpC or the *bla*OXA-51 gene, which provides a string promoter and leads to the overexpression of the genes [55]. Nonetheless, it is likely that several other mutational events remain to be characterized for their association with antibiotic resistance. Unfortunately to date, no genotype-to-phenotype study has been performed. The developers of the ARG-ANNOT ARGs database [26] have looked for ARGs in a collection of 178 *A. baumannii* strains, but they did not compare the output with phenotypic data. 

### 2.7. Stenotrophomonas maltophilia

As for *A. baumannii*, *S. maltophilia* is often met in hospital-acquired infections such as HAP, especially in patients to whom carbapenems have previously been administered. Indeed, *S. maltophilia* is intrinsically resistant to carbapenems, and more globally to all beta-lactams except the ticarcillin-clavulanate association. This phenotype is due to the constitutive expression of two beta-lactamases: L1 (belonging to the Ambler class B) and L2 (belonging to the Ambler class A and being susceptible to the inhibition by clavulanate) [56]. The level of expression of L2 combined to the expression of intrinsic efflux pumps (SmeABC, SmeDEF) can lead to resistance to all beta-lactams [57]. *S. maltophilia* also resists aminoglycosides in a temperature-dependant fashion involving the polarity of the lipopolysaccharide [58,59]. Besides, it remains susceptible to fluoroquinolones even if they bind the DNA gyrase with less efficiency (the serine or threonine usually found in the position 83 of GyrA being a glutamine in *S. maltophilia*) [60]. Of note: unlike other HAP pathogens, resistance to fluoroquinolones in *S. maltophilia* does not seem to arise from mutations in the topoisomerases [60,61]. As for *P. aeruginosa* and *A. baumannii*, inferring the antibiotic susceptibility of *S. maltophilia* from genomic data shall be challenging as the mutational events leading to the overexpression of intrinsic ARGs (especially that of L2 and efflux pumps) remain to be determined. Nonetheless, *S. maltophilia* is susceptible to sulphonamides, and the sulfamethoxazole-trimethoprim combination is recommended as a first-line regimen in infections caused by *S. maltophilia*. As sulphonamide resistance occurs through the acquisition of *sul* genes [62] but also the overexpression of SmeDEF [57], the susceptibility to sulphonamides shall also be difficult to predict with accuracy. 

### 2.8. Staphylococcus aureus

*S. aureus* is a major agent of HAP. Resistance to the main antibiotics used in HAP mostly occur via the acquisition of ARGs. Resistance to penicillins is mediated by the acquisition of the beta-lactamase encoding gene *bla*Z [63] and methicillin resistance arises via the acquisition of the PBP2a—encoding gene *mecA* [64]. Resistance to aminoglycosides in *S. aureus* is due to the acquisition of the *aph(3′)-IIIa, ant(4′)-Ia and aac(6′)-aph(2)’’* genes, while resistance to fluoroquinolones occur through mutations in the topoisomerases [65]. Resistance to glycopeptides is more complex: it can be due to the acquisition of the van operon, but such strains have been rarely isolated to date. More common are strains with intermediate susceptibility to glycopeptides (glycopeptide intermediate *S. aureus*, GISA) due to the thickening of the cell wall [66] through the overexpression of *vraSR*, a two-component system that regulates the expression of *murZ*, *pbp2* and *sgtB* that are involved in the cell wall synthesis [67]. Another gene, *tcaA*, [68], and more recently *yycG* (a component of the WalKR sensory regulatory system) have also has also been associated with the GISA phenotype [69], suggesting that it can be reached by several routes. Still, the precise mutational events in *vraSR*, *tcaA* and *yycG* (and possibly in other genes associated to the GISA phenotype) remain to be determined in order to predict the GISA phenotype from genomic data. Likewise, resistance to linezolid can arise from the acquisition of the *cfr* gene (that encodes an 23S rRNA methyltransferase) [70] and/or by mutations in the 23S rRNA gene. *S. aureus* harbours five copies of this gene, and the linezolid MIC increases along with the number of mutated copies [71]. Hence, recovering five distinct copies of the gene using short reads shall be challenging and likely results in only one assembled, consensus copy of the gene. Hence, the identification of mutations shall require the re-mapping of reads against the consensus copy, or the use of long-reads sequencing methods. We identified four genotype-to-phenotype studies [28,42,43,44]. 

For penicillin resistance, the genomic prediction consists of the detection of the *bla*Z gene. In the Bradley et al. study, a high rate of ME was observed (11.7%), likely because of the lack of sensitivity of phenotypic methods (Becton-Dickinson Phoenix and nitrocefin disks in this study) that served as comparators [43]. Besides, a careful inspection of the *bla*Z sequence revealed in six cases a base insertion or deletion causing a frameshift in the Gordon et al. study [28]. As for methicillin, very good performances were found, the VME being caused by an overexpression of *bla*Z and the ME by a likely low expression of *mecA*. The highest rate of VME was observed for ciprofloxacin (1.2–4.6%, Table 1). While some re-testing revealed that the strains were indeed susceptible, some remained resistant and no explanation could be given [28]. A limited number of gentamicin-resistant strains could be tested in the Gordon et al. and Bradley et al. studies, yet some VME were observed, with no explanation. Conversely, no ME were found. As for vancomycin, no GISA were included in the dataset so that the VME rate could not be assessed. Lastly, no study included linezolid in the panel of tested antibiotics.

## 3. Discussion

As of now, and in line with the recent EUCAST consultation [72], using WGS to infer the antibiotic susceptibility pattern of HAP-causing pathogens requires more studies to fill the current caveats. Indeed, solid data on *E. coli*, *P. aeruginosa*, *S. aureus* and to a lesser extent, *K. pneumoniae* have been published, but there are no published data on the other HAP-causing pathogens such as other Enterobacteriaceae and *A. baumannii*. 

The performances of WGS for inferring the antibiotic susceptibility profiles of *E. coli* and *S. aureus* were high, with few actual discrepancies with conventional methods. Especially, the prediction for first line antibiotics such as methicillin for *S. aureus* and 3GC for *E. coli* was respectively correct in more than 99% and 97% strains. From a CMg perspective, a rapid NGS-based test could hence allow a rapid antibiotic adaptation in case of either resistance or susceptibility of those pivotal antibiotics. We can expect that in species with a similar background such as non-AmpC—producing Enterobacteriaceae, WGS shall predict antibiotic susceptibility within the same range of accuracy. As for AmpC-producing Enterobacteriaceae, the prediction of 3GC susceptibility shall be tricky given that the mutational events leading to the overexpression of AmpC are barely known. Still, for these Enterobacteriaceae, a fourth-generation cephalosporin (cefepime) that resists AmpC hydrolysis should be considered in the first line of treatment. Cefepime resistance occurs through the acquisition of ESBLs; therefore, the correct prediction rates for cefepime susceptibility should be high. Nonetheless, we shall expect some difficulties to infer the phenotype when several combined mechanisms can lead to the resistance of one given antibiotic against which they cannot individually confer resistance. For instance, carbapenem resistance in *K. pneumoniae* can result from the production of ESBL and/or acquired AmpC together with a loss of porin [73]. 

Even more complex is the situation in *P. aeruginosa*. Indeed, results were not as good for meropenem, amikacin and levofloxacin, likely due to the overexpression of the various chromosomal efflux pumps or other unexpected mechanisms [74,75,76]. The diversity of genetic events together with the possible acquisition of ARGs make genotype-to-phenotype prediction in *P. aeruginosa* extremely difficult. As of now, the precise set of mutations associated with the expression of the efflux pumps is not known. In this case, bioinformatic tools such as machine learning shall be used for a high number of isolates in order to associate mutational events to a resistant phenotype [51] and/or the use of transcriptional data to correlate the expression of genes with the phenotype shall be undertaken [74]. Such approaches could also be useful to predict the antibiotic susceptibility profiles of *A. baumannii* and *S. maltophilia*. Still, we acknowledge that predicting with a high accuracy the phenotype from genotype data for these bacteria shall not be possible in the short term with conventional CMg so that probabilistic therapy shall still be used when these bacteria are detected. 

CMg adds even more complexity than WGS as it raises the issue of linking ARGs to their hosts. While some ARGs are borne in the chromosomes of HAP-causing pathogens, several are borne on mobile genetic elements that are commonly shared among these bacteria. Hence, in the case of polymicrobial samples, linking an ARG with a pathogen remains speculative at best. In a CMg study on bone and joint infections samples, we tried to use the respective depths of sequencing of ARGs and contigs from pathogens to infer some connections (i.e., whether a pathogen would harbour an ARG; the depth of sequencing of the ARGs should not be lower than the median depth of sequencing of the contigs from the pathogens) but this approach was proven inaccurate, suggesting that only a fraction of the bacterial population of one given species could carry the ARG [12]. Linking mutational events shall be easier, since they occur in chromosomal genes that can be identified from a given species. Hence, in CMg for polymicrobial samples, it shall be difficult to assess the individual antibiotic susceptibility profiles, and the current way shall be to consider a comprehensive antibiotic susceptibility profile of the bacteria present in the sample including all the ARGs, mutational events and intrinsic phenotypes [12]. Moreover, the genomes of pathogens must be re-assembled enough to detect all the possible ARGs and mutational events linked to antibiotic resistance.

## 4. Conclusions

In conclusion, the translation genotype-to-phenotype appears to be present in some HAP pathogens such as *E. coli* and *S. aureus*. More data are expected for other Enterobacteriaceae, and new approaches are needed for *P. aeruginosa*, *A. baumannii* and *S. maltophilia*. Meanwhile, CMg data on these pathogens should be carefully interpreted. 

## Figures and Tables

**Table 1:** Summary of the performances of the genotype-to-phenotype studies performed on *Escherichia coli*, *Klebsiella pneumoniae*, *Pseudomonas aeruginosa* and *Staphylococcus aureus*. N: number of tested strains. 3GC: third-generation cephalosporins. C: correct, (WGS agrees with conventional methods). ME: major errors (WGS predicts resistance while the strain tested susceptible). VME: very major errors (WGS predicts susceptibility while the strain tested resistant). NT: not tested. NR: not relevant.

Species	*Escherichia Coli*				*Klebsiella Pneumoniae*		*PseudomonaS Aeruginosa*	*Staphylococcus Aureus*				
Reference	Zankari [33]		Stoesser [34] ^a^	Tyson [35]	Stoesser [34] ^a^	Ruppé [40] ^b^	Kos [41]	Köser [42]	Gordon [28] Derivation set	Gordon [28] Validation set	Bradley [43]	Lee [44]
*N*	48		74	76	69	18	388	14	501	491	501	13
Origin	Danish pigs		Bloodstream infections	Cattle	Bloodstream infections	Infection and intestinal carriage	Infections	Infections and nasal carriage	Infections and nasal carriage	Infections and nasal carriage	Infections and nasal carriage	Infections
ARG Database	Resfinder		In house	In house	In house	In house	In house	In house	In house	In house	In house	ARG-ANNOT
Mutation Analysis	None		*ampC*, topoisomerases	*ampC,* topoisomerases	Topoisomerases	Topoisomerases, porins	Topoisomerases, *oprD*	None	Topoisomerases	Topoisomerases	Topoisomerases	Topoisomerases
Ampicillin/Amoxi-Cillin/Penicillin	C	100	98.6	100	NR	NR	NR	100	99.2	94.3	88	NT
	ME	0	1.4	0	NR	NR	NR	0	0.8	5.1	11.7	NT
	VME	0	0	0	NR	NR	NR	0	0	0.6	0.3	NT
Methicillin	C	NT	NT	NT	NT	NT	NT	100	99.6	99.2	100	100
	ME	NT	NT	NT	NT	NT	NT	0	0.2	0.4	0	0
	VME	NT	NT	NT	NT	NT	NT	0	0.2	0.4	0	0
Co-Amoxiclav	C	NT	100	100	98.6	100	NR	NT	NT	NT	NT	NT
	ME	NT	0	0	1.4	0	NR	NT	NT	NT	NT	NT
	VME	NT	0	0	0	0	NR	NT	NT	NT	NT	NT
3GC	C	100 ^c^	97.2 ^d^	98.7 ^d^	97.2 ^d^	100	NT	NT	NT	NT	NT	NT
	ME	0 ^c^	1.4 ^d^	0 ^d^	1.4 ^d^	0	NT	NT	NT	NT	NT	NT
	VME	0 ^c^	1.4 ^d^	1.3 ^d^	2.9 ^d^	0	NT	NT	NT	NT	NT	NT
Carbapenems	C	NT	100 ^e^	NT	98.6 ^e^	100	92.4 ^e^	NT	NT	NT	NT	NT
	ME	NT	0 ^e^	NT	0 ^e^	0	3.7 ^e^	NT	NT	NT	NT	NT
	VME	NT	0 ^e^	NT	1.4 ^e^	0	3.9 ^e^	NT	NT	NT	NT	NT
Fluoroquinolones	C	97.9	100 ^f^	100 ^f^	91.3 ^f^	100	92.8 ^g^	NT	98.0 ^f^	98.0 ^f^	95.2 ^f^	100
	ME	0	0 ^f^	0 ^f^	2.9 ^f^	0	3.1 ^g^	NT	0.6 ^f^	0.2 ^f^	0.2 ^f^	0
	VME	2.1	0 ^f^	0 ^f^	5.8 ^f^	0	4.1 ^g^	NT	1.4 ^f^	1.2 ^f^	4.6 ^f^	0
Amikacin	C	NT	NT	NT	NT	100	81.5	100 ^h^	NT	NT	NT	NT
	ME	NT	NT	NT	NT	0	7.7	0 ^h^	NT	NT	NT	NT
	VME	NT	NT	NT	NT	0	10.8	0 ^h^	NT	NT	NT	NT
Gentamicin	C	100	100	100	98.6	100	NT	100	100	99.6	99.8	100
	ME	0	0	0	0	0	NT	0	0	0	0	0
	VME	0	0	0	1.4	0	NT	0	0	0.4	0.2	0
Tobramycin	C	NT	NT	NT	NT	NT	NT	100	NT	NT	NT	NT
	ME	NT	NT	NT	NT	NT	NT	0	NT	NT	NT	NT
	VME	NT	NT	NT	NT	NT	NT	0	NT	NT	NT	NT
Vancomycin	C	NR	NR	NR	NR	NR	NR	NT	100	100	100	NT
	ME	NR	NR	NR	NR	NR	NR	NT	0	0	0	NT
	VME	NR	NR	NR	NR	NR	NR	NT	0	0	0	NT

^a^. Results after re-testing the phenotype with gradient diffusion are showed here; ^b^. Some strains of the set were clones. All strains were multidrug-resistant isolates; ^c^. Results for cefotaxime; ^d^. Results for ceftriaxone; ^e^. Results for meropenem only; ^f^. Results for ciprofloxacin; ^g^. Results for levofloxacin; ^h^. Results inferred from kanamycin. ME: percentages in bold highlight that less than 10 susceptible strains were tested while; VME: percentages in bold highlight that less than 10 resistant strains were tested.
